# The Patient’s Perspective on Proton Radiotherapy of Skull Base Meningioma: A Retrospective Cross-Sectional Survey

**DOI:** 10.3389/fonc.2022.677181

**Published:** 2022-08-05

**Authors:** Katharina Seidensaal, Jonas Sailer, Semi Ben Harrabi, Johannes von Gehlen, Irina Seidensaal, Fabian Weykamp, Denise Bernhardt, Jürgen Debus, Klaus Herfarth

**Affiliations:** ^1^ Department of Radiation Oncology, Heidelberg University Hospital, Heidelberg, Germany; ^2^ Heidelberg Institute of Radiation Oncology (HIRO), Heidelberg, Germany; ^3^ National Center for Tumor diseases (NCT), Heidelberg, Germany; ^4^ Heidelberg Ion-Beam Therapy Center (HIT), Department of Radiation Oncology, Heidelberg University Hospital, Heidelberg, Germany; ^5^ Clinical Cooperation Unit Radiation Oncology, German Cancer Research Center (DKFZ), Heidelberg, Germany; ^6^ Department of Business Psychology, Fachhochschule für Ökonomie und Management (FOM), Munich, Germany; ^7^ Rehabilitation facility for mentally ill and disabled (ERPEKA), Nuremberg, Germany; ^8^ Department of Radiation Oncology, Klinikum rechts der Isar, Munich, Germany; ^9^ German Cancer Consortium (DKTK), Heidelberg, Germany

**Keywords:** skull base meningioma, proton therapy, cross sectional study, patient report, radiotherapy

## Abstract

**Background:**

Radiotherapy plays an important role in the management of skull base meningioma. The aim of the study was to investigate patient-reported outcomes.

**Methods:**

A questionnaire of 20 items was sent to 192 patients with meningioma of the skull base who have been treated with proton therapy at a single institution. The survey included dichotomous, scaling, and open questions about symptoms, social distancing, rehabilitation, work, reintegration, limitations in recreational activities, as well as daily life activities and correlating diagnoses. Additionally, symptoms were reported retrospectively by the patients at different time points. In total, 128 patients (66.7%) responded. The median age at the time of RT was 55 years (range: 28-91); the majority were female (79%). The median time between the treatment of meningioma and the survey was 38.5 months (range: 7-100).

**Results:**

The most common initial symptoms were visual impairment (*N*=54, 42.2%), dizziness (*N*=38, 29.7%), and double vision (*N*=32, 25%). The most limiting symptom in daily life at the time of the survey was fatigue (*N*=31, 24.2%); a significant proportion of patients reported depression as associated with diagnosis (31.3%). Only 53% of patients reported occupational activity before treatment, this number did not increase with time. Only *N*= 40 (31.3%) and *N*=35 (27.3%) patients reported no limitations in daily household chores or recreational activities by the disease and treatment. The course of cognitive function after treatment showed a temporary deterioration with subsequent improvement. Except for the improvement in emotional functioning, most domains showed a temporary deterioration during radiotherapy, still, the values reached after 6 months differed weekly or moderately from the initial values.

**Conclusion:**

Besides neurological deficits, patients with skull base meningioma experience a variety of unspecific symptoms, which can be most limiting in daily life. Even successful treatment does not necessarily translate into the alleviation of those symptoms. A greater focus on the characterization of those symptom complexes is necessary. Greater focus on functional structures such as the hippocampus might improve the results. Due to the retrospective character, this study is hypothesis-generating.

## Introduction

Meningioma belongs to the most common primary brain tumors of the adult and is benign in more than 90% of cases. They can be asymptomatic and display a steady size for a long period of time and observation by serial high-resolution magnetic resonance imaging can be an option, especially for small tumors (1). In case of neurological symptoms or continued growth, treatment options include surgery or radiation therapy (2). Excellent local control and survival rates have been reported for both options previously (3, 4), however, a prospective comparison is lacking and the optimal treatment strategy is under debate. Conservation of function is gaining increasing importance and the frequency of surgical management has decreased from 2004 to 2012 toward an increase in observation (5).

Skull base meningiomas arise at the petroclival region, sphenoidal wing, or the cavernous sinus. Due to the complex anatomy of the skull base, involvement of vital structures such as cranial nerves or blood vessels is common. Complete resection with an acceptable rate of morbidity is rarely an option in these locations. Subtotal surgery of more extensive tumors and preservation of involved cranial nerves may reach fast decompression of critical structures (e.g., brain stem, optic nerve, or temporal lobe) and improve neurological function. Radiotherapy alone or after subtotal surgery is indicated for many patients with skull base meningiomas. Radiosurgery is possible in many cases, but larger, ill-defined tumors and those that involve radiosensitive structures such as the optic nerve are optimally treated with normofractionated radiotherapy (6).

The diagnosis of skull base meningioma can base solely on MRI; in a previously reported series of patients treated by radiotherapy this applied to approximately 38% of cases (3). When the risk of biopsy is inacceptable or the patient rejects the procedure, ^68^Ga-DOTATOC PET/CT with a radiolabeled somatostatin analogue can be performed before radiotherapy to support the diagnosis. Especially at the skull base, DOTATOC PET/CT has a higher sensitivity than MRI and can support target volume delineation (7).

Depending on the location of a meningioma, patients can experience neurological or cognitive deficits, headaches, or fatigue that influence the patient’s quality of life. Since treatment can also affect function and quality of life, patient reported outcomes are important to understand treatment effectiveness and acute and late toxicity. Herein we present a survey of meningioma patients treated with proton radiotherapy at a single institution.

## Methods

We designed a questionnaire with 20 items, which was sent to 192 patients who have been consequently treated at our institution for meningioma of the skull base with protons. Patients with histologically confirmed WHO grade II and III meningiomas were excluded as the target volume and radiation dose concept are different for higher grade histology. Cases were included if treatment was performed according to concepts for low-grade meningioma without previous histological confirmation. Clinical decision-making in no biopsy-proven cases was guided by the morphological appearance on MRI and DOTATOC uptake of the tumor. The minimum follow-up after the end of radiotherapy of the contacted patients was 6 months.

The questionnaire consisted of 20 items, including multiple choice questions, dichotomous questions, scaling questions, and open-ended questions. Scaling questions were especially used to grade symptoms (1=not at all, 2=slightly, 3=moderately, 4= very, 0= I don’t know) and consisted of 29 questions, which were inspired by the European Organization for Research and Treatment of Cancer Quality of Life Questionnaires EORTC QLQ-30 and EORTC-BN20 questionnaires (8–10). Symptoms were reported as remembered by the patient at four following time points, before radiotherapy (T1), at the end radiotherapy (T2), 6 months after radiotherapy (T3), and in the last week before survey (T4). Inquiry about social distancing, rehabilitation, work, reintegration into the working life, and the correlating diagnoses depression and stroke was performed by dichotomous questions (yes/no) and combined with open-ended questions. Inquiry about limitations in recreational activities (hobbies, sports) as well as daily life activities (e.g., household chores) was performed by scaling questions. Furthermore, patients were asked to describe the initial symptoms that led to the diagnosis (multiple choice combined with open question) and to state which side effects affected them most in daily life (open question).

### Patient Characteristics

The response rate of the survey was 66.7% (*N*=128 patients). The median age at the time of radiotherapy was 55 years (28-91); the vast majority of patients were female *N*=101 (79%). The median time between the treatment of meningioma and the survey was 38.5 months (7-100).

The represented meningioma locations were graded according to the predominantly involved location as follows: sphenopetroclival *N*=91 (71.1 %), orbit/optical nerve *N*=14 (10.9%), petroclival *N*=8 (6.3%), petrosal anterior *N*=6 (4.7%), olfactory nerve *N*=3 (2.3%), frontobasal *N*=1 (0.8%), cerebellopontine angle *N*=1 (0.8%), foramen magnum *N*=2 (1.6%), multiple lesions *N*=1 (0.8%) and petrosal posterior *N*=1 (0.8%). Localization was classified according to previous publications (3, 11). Involvement of critical structures was the common factor of those locations and guided decision toward radiotherapy and against surgery after interdisciplinary case discussion. In total, 86 patients had a histologically confirmed benign meningioma (WHO grade I) after previous surgery or biopsy. All patients had a macroscopic tumor on MRI. Radiotherapy was not performed directly after surgery, but when renewed tumor growth was detected in the course of serial MRI follow-up scans in most cases. Of all patients, *N*=42 (31%) had no previous surgery (**Table 1**).

**Table 1:** Patient characteristics.

	n	Range or %
**N**	128	77.6
**Gender**		
Male	27	21
Female	101	79
**Age at Treatment** (median, range)	55	28 – 91
**Timeframe end of treatment-response** in months (median, range)	38.5	7 - 100
**Localization (**n, percent)		
Sphenopetroclival	91	71.1
Orbital/N. opticus	14	10.9
Petroclival	8	6.3
Petrosal anterior	6	4.7
Olfactory nerve	3	2.3
Frontobasal	1	0.8
Cerebellopontine angle	1	0.8
Multiple lesions	1	0.8
Foramen magnum lesion	2	1.6
Petrosal posterior	1	0.8
**DOTATOC diagnostic** (n, percent)		
Yes	51	39.8
No	77	60.2
**Thereof DOTATOC positive** (n, percent)		
Yes	51	100
**Treatment** (n, percent)		
histologically confirmed	86	67.2
Biopsy	6	4.7
(Partial) resection	81	63.3
Not histologically confirmed	42	32.8
No previous surgery	41	32
**Number of foregoing resections**	n	%
0	42	32.8
1	59	46.1
2	19	14.8
3	6	4.7
4	2	1.6

### Treatment Details

Treatment was performed at Heidelberg Ion-Beam Therapy Center from October 2010 to September 2018. Proton radiotherapy was performed by the method of active scanning. The medium planning target volume (PTV) was 45.3 ccm (3.96-459.8 ccm, IQR 52.74). To create the PTV, a 3 mm margin was added to the GTV.

### Statistical Evaluation

Variables describing the symptoms before and after radiotherapy at four time-points (T1-T4) were combined into seven domains and tested for reliability by Chrombach’s alpha. In the scope of this work, we did not separate symptoms of disease and toxicities strictly. Symptom complexes also include possible toxicities as in the case of side effects of skin and mucosa or gastric symptoms:

1. Fatigue included variables (*N*=2) addressing tiredness and weakness.
2. Gastric symptoms included variables (*N*=3) addressing nausea, vomiting, and loss of appetite.
3. Cognitive function included variables (*N*=5) addressing impaired concentration, forgetfulness, difficulties in learning, difficulties in comprehending language, and difficulties in writing.
4. Emotional function included variables (*N*=4) addressing having many sorrows, feeling very tense or irritable, and sadness.
5. Neurological impairment included variables (*N*=8) addressing anosmia/dysgeusia, auditory or visual impairment, double vision, vertigo, disorientation, gait impairment, and tactile perception.
6. Side effects of skin and mucosa included variables (*N*=5) addressing alopecia, erythema, hyperpigmentation/depigmentation, xerostomia, and xerophtalmia.
7. Pain included variables (*N*=2) addressing headaches and being limited in daily life by pain.

For the analyses of the temporal changes of the symptom domains, single factor variance analyses with repeated measurement were carried out. When the F-Test of the ANOVA (global test) was significant, *post-hoc* T-Tests with Bonferroni adjustment were performed for pairwise comparisons. As T4 was differing between the individual patients, we performed correlation analyses between the timeframe of survey and the end of radiotherapy (Spearman’s rho).

### Treatment Response Analysis

Survival analysis for progression-free survival (PFS) was performed by the method of Kaplan-Meier. Response analysis was performed according to Response Evaluation Criteria in Solid Tumors (RECIST 1.1). The measurements were performed in two transversal directions on the planning MRI scan and the most current follow-up MRI. Analysis was performed by SpSS v. 25.

### Dosimetric Analysis

Twenty patients were systematically chosen for dosimetric analysis based on the PTV size. The sample represented the size distribution of the whole cohort. Additional organs at risk (OAR) were manually delineated. In total, each dataset included the following OAR: Eye bulb, optic nerve, chiasm, pituitary gland, inner ear, parotid gland, brain stem, spinal cord, temporal lobe, hippocampus, amygdala, mammillary body, subvetricular zone, thalamus, hypothalamus, frontal lobe, and carotid artery. Paired organs were considered as ipsilateral or contralateral to the tumor mass.

## Results

### Initial Symptoms and Associated Diagnoses

Initial symptoms reported by the patients included visual impairment (*N*=54, 42.2%), dizziness (*N*=38, 29.7%), double vision (*N*=32, 25%), headaches (*N*=30, 23.4%), exophthalmos (*N*=29, 22.7%), unsteady gait (*N*=29, 22.7%), hearing impairment (*N*=14, 10.9%), and facial pain (*N*=13, 10.2%). Thirty-two patients reported that the meningioma was an incidental finding (25%). In the additional open questions several symptoms were added; the most common were in eight patients (6.3%) paresthesia, in five paresis/paralysis (4%), and dysgeusia/anosmia in three (2%). When compared to documented symptoms at first presentation, there were no gross discrepancies.

When asked about associated diagnoses, 40 patients (31.3%) reported that they had a previous history of depression, 84 (65.6%) replied that they have never been diagnosed with depression, while four answers remained missing. A previous history of stroke was reported by 12 patients (9.4%), while 114 (89.1%) reported no previous history (two missing). Interestingly, pituitary gland impairment was reported by 11 patients only (8.6%), while 65 reported no pituitary gland impairment (50.8%), and 42 (32.8%) reported that they have never been tested (10 missing).

### Participation in Working Life and Everyday Activities

Sixty-eight patients (53%) reported that they pursued an occupational activity before treatment; of those, working full-time *N*=30 (23.4%), part-time *N*=38 (29.7%), none-working *N*=57 (44.5%), and three answers were missing. After the treatment, professional activity was reported by 51 patients (39.8%); working full-time were *N*=20 (15.6%), part-time *N*=29 (22.7%), none-working *N*=59 (46.1%) and 18 (14%) answers remained missing. The timeframe until continuation of work was given at a median of 8.67 weeks (0-86.67) by *N*=47 patients (92% of those who resumed work, four missing). Of those who discontinued work, *N*=18 stated that the reason was the meningioma and *N*=3 stated that this was in part due to meningioma. Forty-eight patients (37.5%) reported that they have undergone medical rehabilitation after treatment, while *N*=78 (60.9%) did not (two missing).

When asked about current limitations in daily household chores or recreational activities, only *N*= 40 (31.3%) and *N*=35 (27.3%) reported no limitations, respectively. Social withdrawal was reported by *N*=38 (29.7%) of patients, while *N*=89 (69.5%) reported no social withdrawal (one missing).

The most frequently reported most limiting symptoms in daily life were reported in an open question as fatigue (*N*=31, 24.2%), dizziness (*N*=19, 14.8%), visual impairment (*N*=14, 10.9%), cognitive impairment (*N*=13, 10.2%), headaches (*N*=9, 7%) and unsteady gait (*N*= 8, 6.3%).

There was no correlation of fatigue, depression, or social withdrawal to gender, age at the beginning of radiotherapy, or PTV size (grouped by median) on Chi-square test.

### Temporal Changes of Symptom Domains

The median values of the seven domains ranged between not at all (1) and moderately (3), the highest median value was reached for fatigue (2.81) at T2.

**Figure 1:**
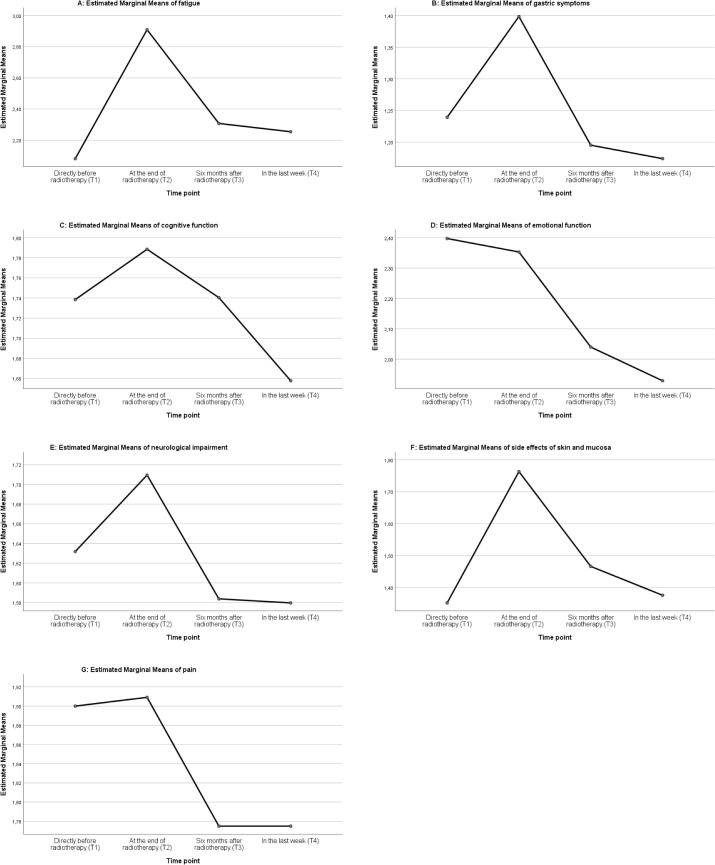
ANOVA Analysis of patients treated by proton radiotherapy: graphic analysis of the seven domains fatigue **(A)**, gastric symptoms **(B)**, cognitive function **(C)**, emotional function **(D)**, neurologic impairment **(E)**, side effects of skin and mucosa **(F)**, and pain **(G)**, at the time points T1-T4.

Variance analysis with repeated measurements showed that the symptom complex fatigue was differing significantly over time. The highest value was reached at the direct end of radiotherapy (T2), which significantly differed from the values before (T1) and ≥ than 6 months after radiotherapy (T3, T4). The value of fatigue decreased after the end of radiotherapy but remained higher at T4 compared to the initial values at T1 (**Figure 1A**, F=35.759, p=.00, partial η^2 =^ 0.242). The Cohen effect size (1988) showed a large effect (*f*=0.565).

There was no significant difference in the patient observed cognition over time (**Figure 1C**, F=2.447, partial η^2 =^ 0.023, p= 0.084).

The symptom complex emotional function showed a steady decrease over time, reflecting a reduction of psychological stress. The difference of the values before and directly after radiotherapy (T1, T2) and ≥ 6 months after treatment (T3, T4) was significant (**Figure 1D**, F=34.327, partial η^2 =^ 0.245, p= 0.000) and the analysis of the effect size showed a large effect (*f*=0.57).

Gastric symptoms defined as nausea and loss of appetite were enhanced at the end of radiotherapy (T2) and significantly different to the other time points (T1, T3, T4 **Figure 1B**, F=17.503, p=.00, partial η^2 =^ 0.138). The effect of this observation was moderate (*f*=0.4).

Side effects of skin and mucosal lining were significantly increased at the direct end of radiotherapy (T2) and decreased after ≥ 6 months (T3, T4) (**Figure 1F**, F=35.040, partial η^2 =^ 0.278, p= 0.000). The analysis of the effect size showed a large effect (*f*=0.621).

Neurologic impairment increased significantly at the end of radiotherapy (T2) compared to the later time points (T3, T4) but the effect size of the observation was small (**Figure 1E**, F=4.677, partial η^2 =^ 0.049, p=0.010, *f*=0.227).

The value of the symptom complex pain seemed to decrease over time (**Figure 1G**), however, the effect size was small (F=3.908, partial η^2 =^ 0.035, p= 0.013, *f*=0.190).

As the time point T4 was different for the individual patients, we performed a non-parametric correlation analysis (Spearman’s rho) between the different domains and the timeframe from the end of radiotherapy to the time point of survey. There was no significant association of the domains with time >6 months.

### Evaluation of Treatment Response on MRI and Analysis of Local Control

**Figure 2:**
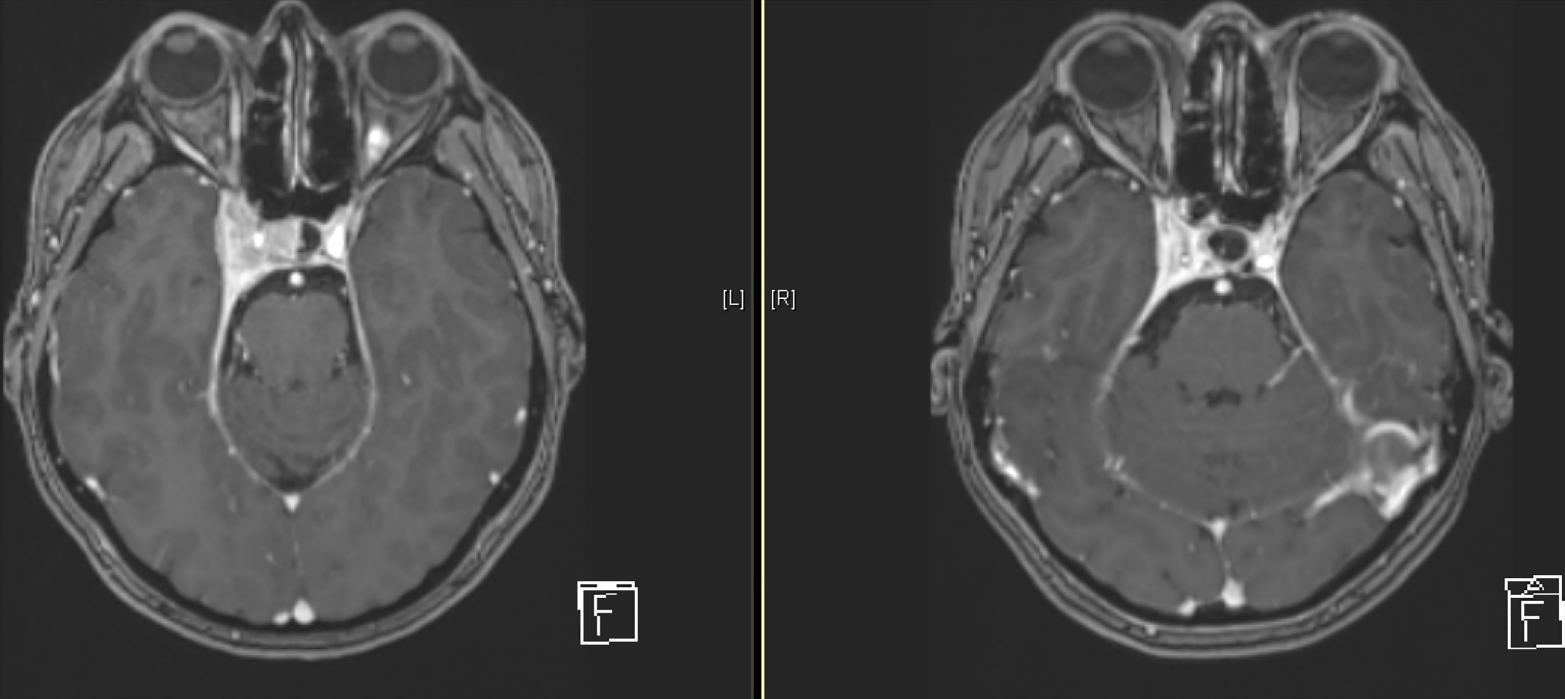
Example of a treated meningioma of the right-sided cavernous sinus at the beginning of treatment (right) and after 8 years of follow-up (left).

The majority of patients showed a stable disease (N= 121, 94.5%) according to RECIST 1.1. Only five patients (3.9%) had a partial remission, there was no complete response, and no progressive disease. The median reduction of the sum of the two diameters was -7.77% (-35.85%-11.36%). When considering the long and short diameter separately, a stronger reduction was seen for the short diameter with -9.52% (-83.33% - 60%) compared to the long diameter -4.76% (-43.75% – 13.33%) (**Figure 2**).

Additionally, we performed volumetric analysis for 20 consecutive patients of the cohort and observed a stronger volume reduction compared to RECIST of -24% (-47% -48%), in absolute values the median reduction was -2.9ml (-30.58ml – 0.27ml). The according reduction of the long diameter was -5.71% (-30.77% - 6.25%), for the short diameter -13.39% (-60.71% - 12.5%) and for the sum of the two diameters -8.33% (-32% – 2.04%).

In four cases, growth of the meningioma was detected and lead to initiation of further treatment, however, this growth did not meet formal RECIST 1.1 criteria (**Table 2**). As a result, the estimated progression-free survival probability in 2 years was 99.2% and in 5 years 97.6%.

**Table 2:** Treatment characteristics.

	Median	IQR	Range
**PTV ccm**	45.265	52.735	3.96 – 459.77
**Treatment mode**	n		Range or %
Active scanned protons	128		100
**Dose specification**			
	Median	IQR	Range
Total dose Gy(RBE)	54	0	50-58
Number of fractions	30	1.75	25-32
Dose of single fraction	1.8	0.150	1.8-2
**Outcome (RECIST 1.1)**	n		%
CR	0		0
SD	121		94.5
PR	5		3.9
PD	0		0
Growth without fulfilling RECIST criteria	4		3.1
Follow-up MRI missing	2		1.6

### Dosimetric Parameters

When considering dose to functional neuronal structures, we observed an *D_2_* of 47.35 GyRBE and a *D_50_* of 27.64 GyRBE to the ipsilateral and *D_2_* of 12.69 GyRBE and *D_50_* of 6.19 GyRBE to the contralateral hippocampus. The *D_2_* to the ipsilateral and contralateral hypothalamus, temporal lobe, thalamus, amygdala, and subventricular zone were 43.50/39.44 GyRBE, 51.55/14.37 GyRBE, 18.14/7.57 GyRBE, 51.39/14.19 GyRBE, 33.88/11.72 GyRBE, respectively. For more details, please refer to **Tables 3**, **4**.

**Table 3:** Near maximal dose to organs at risk.

	Laterality	D2%, mean	D2% Standard deviation
PTV		55.65	0.44
Chiasm		48.15	7.02
Brainstem		47.28	9.44
Spinal cord		0.50	0.67
Whole brain-PTV		37.72	6.87
Amygdala	contralateral	14.19	17.92
Amygdala	ipsilateral	51.39	2.97
Inner ear	contralateral	8.48	14.56
Inner ear	ipsilateral	40.59	13.27
Optic nerve	contralateral	26.37	17.38
Optic nerve	ipsilateral	45.17	12.67
Temporal lobe	contralateral	14.37	15.78
Temporal lobe	ipsilateral	51.55	4.06
Thalamus	contralateral	7.57	8.68
Thalamus	ipsilateral	18.14	11.59
Hippocampus	contralateral	12.69	16.55
Hippocampus	ipsilateral	47.35	9.19
Hypothalamus	contralateral	39.44	12.72
Hypothalamus	ipsilateral	43.50	11.12
Subventricular zone	contralateral	11.72	10.22
Subventricular zone	ipsilateral	33.88	6.59
Frontal lobe	contralateral	14.81	16.57
Frontal lobe	ipsilateral	34.66	16.04
Mammillary body	contralateral	30.43	14.91
Mammillary body	ipsilateral	37.57	12.85
Carotid artery	contralateral	34.50	15.37
Carotid artery	ipsilateral	54.95	1.84

**Table 4:** Median dose to organ at risk.

	Laterality	D50% mean	D50% Standard deviation
PTV		53.47	0.34
Pituitary gland		41.45	12.75
Eye lens	contralateral	0.23	0.56
Eye lens	ipsilateral	1.08	2.82
Lacrimal gland	contralateral	1.25	4.57
Lacrimal gland	ipsilateral	4.37	10.63
Parotid gland	contralateral	0.06	0.27
Parotid gland	ipsilateral	0.41	0.83
Hippocampus	contralateral	6.19	8.54
Hippocampus	ipsilateral	27.64	13.84
Hypothalamus	contralateral	30.70	15.27
Hypothalamus	ipsilateral	36.55	13.12
Subventricular zone	contralateral	0.47	0.94
Subventricular zone	ipsilateral	3.70	3.39
Carotid artery	contralateral	11.16	15.60
Carotid artery	ipsilateral	43.03	13.95

## Discussion

Treatment of skull base meningiomas remains complex and although good long-term survival can be reached for a majority of patients, the potential for tumor- and treatment-related morbidity is significant. The clinical course of those patients can be characterized by repetitive debilitating treatments and long-term impairment of function and quality of life. Twenty-one percent of this cohort had more than one attempt of resection before being admitted to radiotherapy.

When asked about initial symptoms, patients most commonly reported neurological deficits as visual impairment, double vision, and dizziness. Interestingly, not those neurological deficits but fatigue was reported as the most debilitating symptom in daily life. In the longitudinal analysis there was an increase of fatigue after RT with a significant decrease after 6 months, but the difference to the initial levels persisted. Dose to surrounding healthy brain tissues has been shown to increase fatigue, and hippocampal avoidance has been shown to reduce fatigue significantly in patients treated with whole brain irradiation (12). Greater fatigue values were reported for patients with head and neck squamous cell carcinoma treated with IMRT compared to 3D-CRT, which were interpreted as a result of higher mean radiation doses to the posterior fossa (13).

Fatigue is reported to frequently affect meningioma patients in general and not only those treated by radiotherapy. Greater fatigue than the normative population was reported for meningioma patients after surgery even after 108 months (14). In an additional study, significantly more patients reported fatigue before and 1 year after surgery of a WHO grade I meningioma compared to a normative population (15). Thus, the actual cause of fatigue remains to be understood. In a population-based, case-control study, 11% of 1722 patients were treated by radiotherapy and reported statistically lower scores of the domain vitality compared to those who did not receive radiotherapy. However, it should be noted that only patients with inoperable, incompletely resected, or biologically more aggressive tumors received radiotherapy, so attribution of symptoms to RT in this cohort is difficult. Nonetheless, in the time period close to RT, fatigue was reported as a significant side effect, similar to our cohort (16). Our results show that even after successful treatment of meningioma, no alleviation from fatigue can be awaited. A moderate deterioration is possible on the long-term and a significant temporary deterioration at the end of radiotherapy. Similarly, it is known that fatigue can prevail for a long time after successful treatment of a malignant disease. There is no standardized treatment protocol for fatigue, however, treatment possibilities are often underestimated. Promising results have been reached with sports programs improving physical fitness and activity (17). Additionally, individually adapted behavioral-therapeutic measures significantly reduced fatigue values with a lasting effect (18–20). Our work shows that a greater focus should be placed on fatigue and standardized measures for early recognition and treatment need to be developed in the future.

About one-third of patients reported depression as associated with diagnosis, which is significantly higher compared to the prevalence of depressive symptoms among adults in Germany (e.g., age 50-59 years in total 8.4%, among females 10.4%) (21). Approximately the same number of patients reported social withdrawal. Psychological stress and reduced emotional functioning were reported before and at the end of radiotherapy. The values, however, decreased 6 months after RT. The reduction over time was significant and might reflect a feeling of relief after treatment and first follow-up investigations. The aforementioned HRQoL study showed reduced emotional function compared to a normative population within the first 12 months after surgery as well as significant impairment in sleep even after more than 120 months (14). The data suggest that meningioma patients should be screened for signs of depression. Psychological counseling should be offered early in treatment.

Only 53.1% of patients reported professional activity before and 39.8% after radiotherapy. Forty percent of those working before RT reported that cessation of professional activity was completely or partially due to meningioma and treatment. A similar difference was shown for surgery by a Swedish nationwide registry-based matched cohort study. Here, 57% of meningioma patients were working 2 years after surgery compared to 79% before surgery (22). In an additional smaller cohort, 86% were able to return to work after meningioma surgery (23). The differences in the rates before treatment (RT or surgery) most probably reflect the negative selection bias of those referred to radiotherapy. Additionally, a significant portion of patients reported struggles with daily household chores or recreational activities. However only 37.5% received medical rehabilitation. As a rule, all meningioma patients are able to ambulate during RT without treatment interruptions or severe acute complications. Our results show that a stronger focus on medical rehabilitation as well as supportive care might be nonetheless necessary. Limitations can prevail despite successful treatment. A higher reintegration into the labor market would be the goal.

Dose-dependent correlations between structures of the brain such as temporal lobe, frontal lobe, and hippocampi have shown to correlate to neurocognitive impairment in a cohort of patients treated for sinonasal cancer (24). In case of skull base meningioma, the temporal lobe including the hippocampus region is of high interest due to its close location. *Via* functional imaging, it was demonstrated that the compartments of the temporal lobe are responsible for memory and visuospatial, emotional, visual, and sematic processing (25, 26). It is known from whole brain radiotherapy (WBRT) of patients with disseminated brain metastases that sparing the hippocampus region can lead to significantly less cognitive function failure and less fatigue; the here introduced *D_max_* was 16 Gy and *D_100_* 9 Gy (12, 27). In our cohort, we observed a mean near maximum Dose *D_2_* for the ipsilateral hippocampus of 47.35 GyRBE and for the contralateral hippocampus of 12.63 GyRBE, the ipsilateral exceeds the dose known from hippocampus avoidance WBRT. Whether those constraints can be transferred to meningioma patients remains to be answered. For the amygdalae, which is critical for processing emotions and creating and storing memory, dose-dependent volume loss of 0.17% per 1 Gy was shown for patients with primary brain tumors (28). Reduced volumes have been associated with depression by some authors, while others report enlargement during acute depression (29, 30).

Proton therapy has steeper lateral dose gradients which lead to lower medium and low dose to surrounding healthy tissues compared to IMRT or other photon techniques (31). Developing constraints for functional neuronal structures and optimizing RT plans in order to spare those might lead to less long-term effects in meningioma patients, and less subjectively perceived limitations.

We observed high local control rates over 90% and the best response assessed on MRI was stable disease. Considering that the inactivated tumor does not decrease in size, the involvement of critical structures and the pressure applied to surrounding tissues lingers, which might explain the absence of symptom relief. However, significant impairments on domains of quality of life, fatigue, and insomnia reduced global quality of life and considerable risk for long-term sick leave have been reported after meningioma surgery, thus absence or presence of the tumor mass cannot be the only contributing factor (14, 22).

So far, few studies have investigated the patient’s view on the disease and the associated morbidity. Particularly, there is little data on longitudinal changes. Most studies compare treatment modalities and concentrate on tumor, treatment, or patient-specific characteristics which may predict poor quality of life or outcome such as tumor size, associated epilepsy, or higher tumor grade. These parameters are useful for prognostication and treatment decision finding. It is known that the patient’s perception can significantly differ from the physician’s view when symptoms and severity of side effects are reported and graded (7). When acquiring longitudinal data, it has been shown that adherence to repetitive questioning is difficult; in a prospective longitudinal cross-sectional study of patients with meningioma after surgery, 62% of patients completed the questionnaire only once and an additional 24% only twice (14). We decided, therefore, to perform a cross-sectional study and to evaluate symptoms and side effects as experienced and retrospectively taken stock of by the patient. The biggest limitation of this approach is that memory or review errors can lead to a cognitive distortion of the longitudinal data. Further limitations lie in the retrospective character of the investigation. This work does not offer a strict causal allocation of symptoms to disease or treatment but aims to give a more complete picture and is strictly hypothesis-generating.

## Conclusion

Besides neurological deficits, patients with skull base meningioma experience a variety of unspecific symptoms which can be limiting in daily life, as for example fatigue which was reported as most debilitating in our cohort. Even successful treatment does not necessarily translate into alleviation of those symptoms; many patients do not return to work and have limitations in their daily activities. A greater focus on the characterization and understanding of those symptom complexes is necessary as well as finding the causality to disease and treatment. Greater focus on functional structures such as the hippocampus in radiotherapy plan optimization might improve the results.

## Data Availability Statement

The raw data supporting the conclusions of this article will be made available by the authors, without undue reservation.

## Ethics Statement

The studies involving human participants were reviewed and approved by Ethics Committee Medical Faculty University Heidelberg. The patients/participants provided their written informed consent to participate in this study.

## Author Contributions

KS, JS, DB, JD, and KH contributed conception and design of the study; KS, JS, and KH provided the data; KS, JS, SH, and FW analyzed the data; KS, JS, and KH wrote the manuscript; All authors contributed to manuscript revision, read and approved the submitted version.

## Funding

For the publication fee we acknowledge financial support by Deutsche Forschungsgemeinschaft as well as by Heidelberg University.

## Conflict of Interest

The authors declare that the research was conducted in the absence of any commercial or financial relationships that could be construed as a potential conflict of interest.

## Publisher’s Note

All claims expressed in this article are solely those of the authors and do not necessarily represent those of their affiliated organizations, or those of the publisher, the editors and the reviewers. Any product that may be evaluated in this article, or claim that may be made by its manufacturer, is not guaranteed or endorsed by the publisher.

## References

[1] Goldbrunner R Minniti G Preusser M Jenkinson MD Sallabanda K Houdart E. EANO guidelines for the diagnosis and treatment of meningiomas. Lancet Oncol (2016) 17(9):e383–91. doi: 10.1016/S1470-2045(16)30321-7 27599143

[2] Combs SE Ganswindt U Foote RL Kondziolka D Tonn J-C. State-of-the-art treatment alternatives for base of skull meningiomas: complementing and controversial indications for neurosurgery, stereotactic and robotic based radiosurgery or modern fractionated radiation techniques. Radiat Oncol (London England). (2012) 7:226. doi: 10.1186/1748-717X-7-226 PMC3551826 23273161

[3] El Shafie RA Czech M Kessel KA Habermehl D Weber D Rieken S. Clinical outcome after particle therapy for meningiomas of the skull base: toxicity and local control in patients treated with active rasterscanning. Radiat Oncol (London England). (2018) 13(1):54. doi: 10.1186/s13014-018-1002-5 PMC5870393 29587795

[4] Pechlivanis I Wawrzyniak S Engelhardt M Schmieder K. Evidence level in the treatment of meningioma with focus on the comparison between surgery versus radiotherapy. A review. J neurosurgical Sci (2011) 55(4):319–28. 22198584

[5] Agarwal V McCutcheon BA Hughes JD Carlson ML Glasgow AE Habermann EB. Trends in Management of Intracranial Meningiomas: Analysis of 49,921 Cases from Modern Cohort. World neurosurgery. (2017) 106:145–51. doi: 10.1016/j.wneu.2017.06.127 28666914

[6] Mendenhall WM Friedman WA Amdur RJ Foote KD. Management of benign skull base meningiomas: a review. Skull Base. (2004) 14(1):53–61. doi: 10.1055/s-2004-821364 16145585 PMC1151672

[7] Jagsi R Griffith KA Boike TP Walker E Nurushev T Grills IS. Differences in the Acute Toxic Effects of Breast Radiotherapy by Fractionation Schedule: Comparative Analysis of Physician-Assessed and Patient-Reported Outcomes in a Large Multicenter Cohort. JAMA Oncol (2015) 1(7):918–30. doi: 10.1001/jamaoncol.2015.2590 26247417

[8] Aaronson NK Ahmedzai S Bergman B Bullinger M Cull A Duez NJ. The European Organization for Research and Treatment of Cancer QLQ-C30: a quality-of-life instrument for use in international clinical trials in oncology. J Natl Cancer Institute. (1993) 85(5):365–76. doi: 10.1093/jnci/85.5.365 8433390

[9] Chow R Lao N Popovic M Chow E Cella D Beaumont J. Comparison of the EORTC QLQ-BN20 and the FACT-Br quality of life questionnaires for patients with primary brain cancers: a literature review. Supportive Care Cancer Off J Multinational Assoc Supportive Care Cancer. (2014) 22(9):2593–8. doi: 10.1007/s00520-014-2352-7 25015058

[10] Osoba D Aaronson NK Muller M Sneeuw K Hsu MA Yung WK. The development and psychometric validation of a brain cancer quality-of-life questionnaire for use in combination with general cancer-specific questionnaires. Qual Life Res an Int J Qual Life aspects treatment Care rehabilitation. (1996) 5(1):139–50. doi: 10.1007/BF00435979 8901377

[11] Ware ML Pravdenkova S Erkmen K Al-Mefty O. Petroclival and Upper Clival Meningiomas I. In: Lee JH, editor. An Overview of Surgical Approaches, vol. p . Meningiomas. London: Springer London (2009). p. 403–14. doi: 10.1007/978-1-84628-784-8_44

[12] Brown PD Gondi V Pugh S Tome WA Wefel JS Armstrong TS. Hippocampal Avoidance During Whole-Brain Radiotherapy Plus Memantine for Patients With Brain Metastases: Phase III Trial NRG Oncology CC001. J Clin Oncol Off J Am Soc Clin Oncol (2020) 38(10):1019–29. doi: 10.1200/JCO.19.02767 PMC7106984 32058845

[13] Nutting CM Morden JP Harrington KJ Urbano TG Bhide SA Clark C. Parotid-sparing intensity modulated versus conventional radiotherapy in head and neck cancer (PARSPORT): a phase 3 multicentre randomised controlled trial. Lancet Oncol (2011) 12(2):127–36. doi: 10.1016/S1470-2045(10)70290-4 PMC3033533 21236730

[14] Nassiri F Price B Shehab A Au K Cusimano MD Jenkinson MD. Life after surgical resection of a meningioma: a prospective cross-sectional study evaluating health-related quality of life. Neuro-Oncology (2019) 21(Supplement_1):i32–43. doi: 10.1093/neuonc/noy152 PMC6347082 30649488

[15] van der Linden SD Gehring K Rutten GM Kop WJ Sitskoorn MM. Prevalence and correlates of fatigue in patients with meningioma before and after surgery. Neurooncol Pract (2020) 7(1):77–85. doi: 10.1093/nop/npz023 32257286 PMC7104880

[16] Benz LS Wrensch MR Schildkraut JM Bondy ML Warren JL Wiemels JL. Quality of life after surgery for intracranial meningioma. Cancer. (2018) 124(1):161–6. doi: 10.1002/cncr.30975 PMC6415762 28902404

[17] Kessels E Husson O van der Feltz-Cornelis CM. The effect of exercise on cancer-related fatigue in cancer survivors: a systematic review and meta-analysis. Neuropsychiatr Dis Treat (2018) 14:479–94. doi: 10.2147/NDT.S150464 PMC5810532 29445285

[18] Gielissen MFM Wiborg JF Verhagen CAHHVM Knoop H Bleijenberg G. Examining the role of physical activity in reducing postcancer fatigue. Supportive Care Cancer. (2012) 20(7):1441–7. doi: 10.1007/s00520-011-1227-4 PMC3360858 21773676

[19] Gielissen MF Verhagen CA Bleijenberg G. Cognitive behaviour therapy for fatigued cancer survivors: long-term follow-up. Br J Cancer. (2007) 97(5):612–8. doi: 10.1038/sj.bjc.6603899 PMC2360364 17653075

[20] Gielissen MF Verhagen S Witjes F Bleijenberg G. Effects of cognitive behavior therapy in severely fatigued disease-free cancer patients compared with patients waiting for cognitive behavior therapy: a randomized controlled trial. J Clin Oncol Off J Am Soc Clin Oncol (2006) 24(30):4882–7. doi: 10.1200/JCO.2006.06.8270 17050873

[21] Busch MA Maske UE Ryl L Schlack R Hapke U. Prävalenz von depressiver Symptomatik und diagnostizierter Depression bei Erwachsenen in Deutschland. Bundesgesundheitsblatt - Gesundheitsforschung - Gesundheitsschutz. (2013) 56(5-6):733–9. doi: 10.1007/s00103-013-1688-3 23703492

[22] Jakola AS Salvesen Ø Bartek J Henriksson R Smits A Gulati S. Return to work following meningioma surgery: a Swedish nationwide registry-based matched cohort study. Neuro-Oncology Practice. (2020) 7(3):320–8. doi: 10.1093/nop/npz066 PMC7274187 32528713

[23] Kalkanis SN Quiñones-Hinojosa A Buzney E Ribaudo HJ Black PM. Quality of life following surgery for intracranial meningiomas at Brigham and Women's Hospital: a study of 164 patients using a modification of the functional assessment of cancer therapy-brain questionnaire. J neuro-oncology. (2000) 48(3):233–41. doi: 10.1023/A:1006476604338 11100821

[24] Sharma MB Jensen K Amidi A Eskildsen SF Johansen J Grau C. Late toxicity in the brain after radiotherapy for sinonasal cancer: Neurocognitive functioning, MRI of the brain and quality of life. Clin Trans Radiat Oncol (2020) 25:52–60. doi: 10.1016/j.ctro.2020.09.003 PMC7530204 33024844

[25] Pascual B Masdeu JC Hollenbeck M Makris N Insausti R Ding S-L. Large-Scale Brain Networks of the Human Left Temporal Pole: A Functional Connectivity MRI Study. Cereb Cortex. (2015) 25(3):680–702. doi: 10.1093/cercor/bht260 24068551 PMC4318532

[26] Baker CM Burks JD Briggs RG Milton CK Conner AK Glenn CA. A Connectomic Atlas of the Human Cerebrum-Chapter 6: The Temporal Lobe. Operative Neurosurg (2018) 15(suppl_1):S245–S94. doi: 10.1093/ons/opy260 PMC6887748 30260447

[27] Gondi V Pugh SL Tome WA Caine C Corn B Kanner A. Preservation of memory with conformal avoidance of the hippocampal neural stem-cell compartment during whole-brain radiotherapy for brain metastases (RTOG 0933): a phase II multi-institutional trial. J Clin Oncol Off J Am Soc Clin Oncol (2014) 32(34):3810–6. doi: 10.1200/JCO.2014.57.2909 PMC4239303 25349290

[28] Huynh-Le M-P Karunamuni R Moiseenko V Farid N McDonald CR Hattangadi-Gluth JA. Dose-dependent atrophy of the amygdala after radiotherapy. Radiotherapy Oncol J Eur Soc Ther Radiol Oncol (2019) 136:44–9. doi: 10.1016/j.radonc.2019.03.024 PMC7041546 31015128

[29] Rosso IM Cintron CM Steingard RJ Renshaw PF Young AD Yurgelun-Todd DA. Amygdala and hippocampus volumes in pediatric major depression. Biol Psychiatry (2005) 57(1):21–6. doi: 10.1016/j.biopsych.2004.10.027 15607296

[30] van Eijndhoven P van Wingen G van Oijen K Rijpkema M Goraj B Jan Verkes R. Amygdala Volume Marks the Acute State in the Early Course of Depression. Biol Psychiatry (2009) 65(9):812–8. doi: 10.1016/j.biopsych.2008.10.027 19028381

[31] Arvold ND Niemierko A Broussard GP Adams J Fullerton B Loeffler JS. Projected second tumor risk and dose to neurocognitive structures after proton versus photon radiotherapy for benign meningioma. Int J Radiat oncology biology physics. (2012) 83(4):e495–500. doi: 10.1016/j.ijrobp.2011.10.056 22285662

